# Inequities in access to life-saving cardiac devices for heart failure: a comparative analysis of Chile and Mexico

**DOI:** 10.1186/s12872-026-05924-4

**Published:** 2026-05-11

**Authors:** Fernando Lanas, Marco A. Alcocer-Gamba, Carlos A. Narváez, Rubén Aguayo, Pablo López, Hugo Verdejo, Judith Riesgo, Federico Levy, Svetlana V. Doubova, Ricardo Perez-Cuevas, Claudio Muratore

**Affiliations:** 1 Servicio de Cardiología, Hospital Dr. Hernán Henríquez Aravena, Región de La Araucanía, Chile; 2 Dirección general, Centro de Estudios Clínicos de Querétaro S.C., Santiago de Querétaro, México; 3 Servicio de Cardiología, Hospital General ISSSTE Tacuba, Ciudad de México, México; 4 Servicio de Cardiología y Arritmias, Hospital San Juan de Dios, Santiago, Chile; 5 Unidad de Hospitalizados CR Cardiovascular, Hospital Dr. Guillermo Grant Benavente, Ciudad de Concepción, Chile; 6 División de Enfermedades Cardiovasculares – Unidad Coronaria, Pontificia Universidad Católica de Chile, Av. Libertador Bernardo O’Higgins 340, Santiago, 8320091 Chile; 7 Latin America Clinical Research and Medical Science department, Medtronic Mexico S RL de CV, Ciudad de México, Mexico; 8 Unidad de Investigación Epidemiológica y Servicios de Salud del CMN SXXI, Instituto Mexicano del Seguro Social, Avenida Cuauhtémoc 330, Colonia Doctores, Delegación Cuauhtémoc, Ciudad de México, 06720 México; 9 Medtronic Argentina, CABA, Argentina

**Keywords:** Heart Failure, Reduced Ejection Fraction, Cardiac Implantable Electronic Device, ACC/AHA clinical guideline, Chile, Mexico

## Abstract

**Background:**

Heart failure (HF) remains a leading cause of death in Latin America, yet access to cardiac implantable electronic devices (CIEDs) is limited. Chile provides HF treatment through the AUGE/GES (Universal Access with Explicit Guarantees in Health) program, while Mexico’s public sector lacks such mechanisms for people without health insurance. This study aims to identify demographic and clinical factors associated with not receiving ICD or CRT-D among HF patients in Chile and Mexico.

**Methods:**

This secondary analysis of the PLASMA study included 246 HF patients eligible for implantable cardioverter-defibrillators (ICDs) or cardiac resynchronization therapy with defibrillator (CRT-D) based on the 2017 ACC/AHA/HRS criteria. Variables included sociodemographic and clinical factors. We performed the Chi-square test and multivariable logistic regression analysis.

**Results:**

Out of 246 eligible patients, 41.1% (*n* = 101) received ICD or CRT-D. The mean age of non-recipients was 64.1 years, compared to 58.5 years for recipients. Device implantation rates were higher in Chile (56%) than in Mexico (21%). Compared to Chile, Mexico had more recipients with NYHA class I (11.4% vs. 50%), even though the 2017 ACC/AHA/HRS criteria recommend ICDs and CRT-Ds mainly for symptomatic NYHA II–III patients, with stricter criteria for NYHA I (such as Left Bundle Branch Block, ≥ 40 days post-myocardial infarction, or QRS ≥ 150 ms with optimal therapy). Notably, Mexican patients had 7.6 times higher odds of not receiving a device compared to Chilean patients (aOR 7.58, 95%CI: 3.21–17.91). Care in public hospitals, older age, and NYHA III–IV were also associated with higher odds of not receiving a device, while ventricular arrhythmia and prior revascularization were associated with a lower likelihood of non-implantation (aOR 0.09, 95%CI: 0.04–0.18, and aOR 0.57, 95%CI: 0.33–0.99, respectively), indicating that these patients were substantially more likely to receive a device. No significant sex-related differences were observed.

**Conclusions:**

There are substantial inequities in access to life-saving cardiac devices between Chile and Mexico: structural and demographic factors, particularly the supply capacity of public hospitals and older age, significantly limit access.

**Supplementary Information:**

The online version contains supplementary material available at 10.1186/s12872-026-05924-4.

## Introduction

Cardiovascular diseases (CVD) remain the leading cause of mortality and disability across Europe [1], Latin America, and the Caribbean [2]. In Europe, the management of heart failure (HF) is embedded in comprehensive, explicit policies that integrate clinical guidelines, national chronic disease programs, and financing mechanisms. The European Society of Cardiology (ESC) promotes standardized recommendations for diagnosis, pharmacological treatment, and the use of devices such as implantable cardioverter defibrillators (ICDs) and cardiac resynchronization therapy with defibrillator (CRT-Ds) [3]. Europe is increasingly using nationally organized HF units and accredited tiered networks, guided by the Heart Failure Association of the European Society of Cardiology programs [4]. Moreover, recent global perspectives emphasize the need to move beyond purely device-based prevention strategies toward integrated approaches that address arrhythmic substrate, heart failure progression, and population-level risk, using multidisciplinary research, policy, and community action [5]. The Lancet Commission on Sudden Cardiac Death highlights that optimizing guideline-directed medical therapy and improving access to advanced therapies should be considered as complementary, not mutually exclusive strategies to reduce arrhythmic mortality [5].

In contrast, most countries in Latin America and the Caribbean (LAC) lack specific national policies for HF. LAC reports describe single- center or individual programs, not country-wide, accredited HF units, due to resource constraints and inequitable access [6]. In the LAC region, HF is an increasingly critical clinical and public health concern. With an estimated prevalence of 1%, a one-year mortality rate of 24.5%, and an in-hospital mortality rate of 11.7%, HF significantly contributes to premature mortality [7]. This burden is exacerbated by three interrelated problems: the absence of comprehensive health policies, persistent disparities in healthcare access and quality, and socioeconomic and sex-related inequalities.

Despite regional efforts to strengthen healthcare for non-communicable diseases, few LAC countries have developed comprehensive HF policies. Most responses are embedded within broader CVD or non-communicable disease frameworks, leading to fragmented implementation, chronic underfunding, and weak monitoring of access, quality, and health outcomes [8]. These gaps reflect the limited recognition of HF’s burden and the socioeconomic impact on national priorities, resulting in inadequate policy design and insufficient service capacity in primary and secondary care [9, 10].

The cumulative result of these weaknesses is inequitable access to evidence-based HF care, particularly in public healthcare systems serving vulnerable populations [11, 12]. Gaps persist in the availability of essential medicines, advanced cardiac procedures, and device implantation, primarily affecting marginalized populations [13]. Access to cardiac implantable devices (CIEDs), such as ICDs and CRT-D, remains limited in most LAC countries, constrained by scarce financing, workforce, and infrastructure [14]. Device implantation is highly variable, ranging from 16 per million in Venezuela to 617 per million in Uruguay [11].

Socioeconomic and gender inequities exacerbate these challenges. Vulnerable groups, including women, the elderly, and rural communities, face barriers to insurance coverage, diagnostics, and advanced therapies. Women are less likely than men to receive device implantation or revascularization despite comparable clinical indications [15, 16]. In LAC, the high HF burden, structural inequities that limit access, and high out-of-pocket health expenditures, along with scarce, fragmented epidemiological data, limit understanding of which vulnerable groups are most excluded from technology. A Chilean review describes difficulty accessing left ventricular assist devices and transplants due to cost and limited evaluation capacity [17]. Still, data from LAC remains scarce.

The increasing awareness of inequities in CVD care in LAC highlights the need for research to explore both individual and system-level barriers to device implantation. Understanding the barriers to accessing CIEDs, such as ICDs or CRT-Ds, is essential for promoting global health equity and enhancing cardiovascular access care. While factors like funding limitations, restrictive indications, limited insurance coverage, and procedural delays have been recognized, the individual-level determinants of CIED access remain poorly understood.

Chile and Mexico are suitable countries for gathering more evidence on access to ICDs or CRT-Ds for patients with HF. Chile has a dual public-private system. The public subsystem (FONASA + National Health Services System, SNSS) covers approximately 80% of the population, mostly lower-income, older, and sicker groups. Private insurers (ISAPRES) cover approximately 20%, mainly higher-income and younger people. This fragmentation results in adverse selection, with private insurers attracting healthier, wealthier individuals, and public insurers serving more vulnerable populations with higher health needs.

Chile’s health system guarantees explicit access to HF treatment under the AUGE/GES program. Pharmacological management aligns with the guidelines of the American Heart Association, the American College of Cardiology, and the Heart Rhythm Society (ACC/AHA/HRS) for CIEDs (Class I indications) [18]. However, only 3 in 10 eligible patients receive an ICD or a CRT-D within a year [19].

Mexico’s public healthcare sector is fragmented among several institutions (Social Security, IMSS-BIENESTAR for the uninsured, and the private sector), leading to marked heterogeneity in the capacity to meet healthcare demand [20]. Social Security has more resources, whereas approximately 60% of the population lacks public healthcare insurance and relies on the Ministry of Health services through the IMSS-BIENESTAR program, which does not guarantee explicit health benefits [21]. IMSS-BIENESTAR faces resource shortages of medicines and medical technologies, including CIEDs [21]. In Mexico, the care process for HF faces challenges. Although pharmacological therapy follows ACC/AHA/HRS guidelines, only 1 in 20 eligible HF patients receives an ICD or CRT-D [22]. As a result, the continuity of care from pharmacological treatment to device implantation is often interrupted due to access barriers.

Table 1 provides a brief description of both countries’ health care systems and public health care coverage. The asymmetries in health care resources between the two countries are evident. Mexico has a lower GDP per capita and allocates fewer financial resources than Chile. This also includes explicit HF coverage and ICD/CRT implants per million per year [11].

**Table 1:** Key healthcare system differences between Chile and Mexico

Variable	Chile	Mexico
Population	19.764 M (2024)	130.861 M (2024)
Gross Domestic Product (GDP) per capita	16.709 (2024)	14.185 (2024)
Health expenditure (% of GDP )*	10.53 (2024)	5.5 (2023)
Out-of-pocket expenditure (% of total health spending)	32%	41–45%
Health system	Dual public-private with 80% public coverage	Highly fragmented (60% uninsured/public mix)
Explicit HF coverage	Yes	No unified national program
ICD/CRT implants per million/year**	150–250 per million	20–40 per million

Sources: * www.worldbank.org﻿** Reference 11: Rojel et al. Heart Rhytm 02. 2024

The PLASMA study (a Spanish acronym for “Probability of Suffering Arrhythmic Death”) was a prospective, multicenter, one-year cohort study conducted to characterize patients with heart failure and reduced ejection fraction in two Latin American countries—Chile and Mexico—and to identify factors associated with arrhythmic risk and access to advanced therapies.

In the present analysis, we aimed to identify demographic and clinical factors associated with not receiving ICD or CRT-D among patients with heart failure and reduced left ventricular ejection fraction (≤ 35%) who met Class I indications according to the 2017 ACC/AHA/HRS guidelines, and to explore potential sex-related disparities in access to these life-saving therapies.

## Methods

We performed a secondary data analysis of the PLASMA study.

The PLASMA study was conducted from March 2021 to December 2022 in Chile and from January 2020 to May 2022 in Mexico. It involved six hospitals in Chile and seven in Mexico. Adults aged 18 or older with heart failure with reduced ejection fraction (≤ 40%) were enrolled (243 patients in Chile and 253 in Mexico). A non-probability convenience sampling method was used, recruiting 34–50 consecutive patients per hospital.

For this secondary data analysis, we included only patients with a left ventricular ejection fraction (LVEF) of < 35% who were eligible for ICD or CRT-D devices based on the 2017 ACC/AHA/HRS Class I criteria (*n* = 141 patients in Chile and *n* = 105 patients in Mexico, totaling 246 patients).

The 2017 ACC/AHA/HRS Class I criteria comprised:

1. Non-Ischemic Dilated Cardiomyopathy patients with New York Heart Association Functional Classification (NYHA) Class II to III, LVEF of 35% or lower, and optimal pharmacological treatment.
2. Ischemic Heart Disease patients with NYHA Class II to III, LVEF of 35% or lower, at least 40 days post-myocardial infarction (MI), and at least 90 days post-coronary artery bypass grafting (CABG), along with optimal treatment.
3. Ischemic Heart Disease patients with NYHA Class I, LVEF of 30% or lower, at least 40 days post-MI, and at least 90 days post-CABG, with optimal pharmacological therapy.
4. These criteria predominantly correspond to primary prevention indications. However, a subset of patients—particularly those with documented ventricular arrhythmias—may also reflect secondary prevention scenarios, which should be considered when interpreting implantation patterns.

The sample size of 246 patients was sufficient to conduct a multivariable regression analysis, based on the rule of thumb of 10 participants per included variable [23]. The multivariable regression included 17 variables; therefore, the minimum required sample size was 170 patients.

### Study variables

In both countries, the study variables were collected using the same predefined questionnaire that gathered information on the following variables:

Sociodemographic baseline characteristics: age (in years), sex (male and female), education level (basic or no formal education, or secondary or higher), area of residence (urban or rural), country (Chile or Mexico), and hospital type where the patient was cared for (private or public).

Clinical history: obesity (Body mass index ≥ 30 kg/m2), hypertension, diabetes, kidney disease, history of cardiac conditions such as myocardial infarction, paroxysmal tachycardia, atrial fibrillation, atrial flutter, other cardiac arrhythmias, history of revascularization (percutaneous or surgical), and NYHA Classification of the stages of heart failure.

CIED-Related variables: cardiac resynchronization therapy (CRT) devices such as defibrillator-based CRT (CRT-D), and pacemaker-based CRT (CRT-P), implantable cardioverter-defibrillators (ICD), and pacemakers (PM).

### Statistical analysis

We conducted a descriptive analysis of participants’ sociodemographic and clinical characteristics. We used percentages for categorical variables and means and standard deviations for continuous variables.

We compared patients with and without ICD and CRT-D, as well as female and male patients, using the Fisher exact test for categorical variables with expected cell counts < 5 and the Chi-square test for the remaining categorical variables.

We used multivariable logistic regression to identify factors associated with a higher probability of not receiving ICD or CRT-D devices. Our modeling strategy for multicountry analysis was based on VanderWeele and Shpitser’s criterion for confounder selection [24]. These authors recommend including all conceptually and clinically relevant covariates to ensure the final model adjusts for even slight confounding (and is not subject to potential p-value hacking).

Therefore, through a literature review, we identified relevant sociodemographic and clinical variables to be included. The reference categories were female sex, secondary or technical education or higher, receiving care in Chile, urban residence, private hospital, without obesity, without a medical diagnosis of hypertension, diabetes, or kidney disease, without a previous myocardial infarction, without atrial arrhythmia or ventricular arrhythmia, without an earlier PTCA or CRM, and NYHA class I. Before performing the multivariable logistic regression, we confirmed the absence of multicollinearity and interactions among the study covariates. Additionally, the standard errors of the regression model were adjusted for clustering by hospital, since the same physician treated participants at each hospital. We used each hospital’s unique ID to adjust the standard errors. All study variables had complete data, with no missing values. The initial step of the analysis involved gathering data and factors from both countries to establish a broad baseline understanding. We then conducted a country-specific sensitivity analysis to examine how these factors varied across different contexts. Furthermore, since receiving a CRT-P or pacemaker may indicate clinical decisions related to unmeasured contraindications to defibrillators, we performed a multinomial logistic regression as a sensitivity analysis. We examined factors linked to both no device and alternative device (pacemaker or CRT-P) implantation, using guideline-concordant ICD/CRT-D implantation as the reference.

A *p*-value < 0.05 was considered statistically significant. The statistical analysis was performed using Stata 14.0.

## Results

A total of 246 patients with heart failure and reduced left ventricular ejection fraction (LVEF ≤ 35%) who met Class I indications for CIEDs were analyzed: 141 from Chile and 105 from Mexico. Overall, 101 patients (41.1%) had received an ICD or CRT-D either at baseline or during follow-up, while 145 (58.9%) were eligible but did not receive the device during the study period. Eligible patients without an ICD/CRT-D were significantly older than those who received one (mean 64.1 ± 12.3 vs. 58.5 ± 13.4 years, *p* = 0.0008). Device implantation was more frequent in Chile than in Mexico (78.2% vs. 21.8% among those who received ICD or CRT-D). Kidney disease was more prevalent among patients without devices (36.6% vs. 17.8%, *p* = 0.001), whereas ventricular arrhythmias were substantially more common among those who received one (27.7% vs. 5.5%, *p* < 0.001). Additionally, previous interventional procedures were more frequent among those who received one (39.6% vs. 27.6%, *p* = 0.048). There were no significant differences between patients with and without ICD or CRT-D by sex, education level, residence, hospital type, or NHYA heart failure stage in the bivariate analysis. Regarding the type of CIED, the most frequent was ICD (65%) in the group that had a CIED at baseline or received one during the follow up (Table 2).

**Table 2:** General characteristics of patients with heart failure, LVEF ≤35% and ICD or CRT-D in Chile and México (*n* = 246)

General characteristics	Patients who had an ICD or CRT-D at baseline or received one during the follow-up year *n* = 101 (41.0%)	Eligible patients without ICD or CRT-D during the follow-up year *n* = 145 (59.0%)	*P* value
Age (years), mean (SD)	58.5 (13.4)	64.1 (12.3)	0.0008
	**n (%)**	**n (%)**	
Sex			
Male	78 (77.2)	104 (71.7)	*p* > 0.05
Female	23 (22.8)	41 (28.3)	
Country			
Chile	79 (78.2)	62 (42.8)	0.0001
Mexico	22 (21.8)	83 (57.2)	
Residence			
Urban ​​	89 (88.1)	127 (87.6)	*p* > 0.05
Rural	12 (11.9)	18 (12.4)	
Formal education			
Basic or none	75 (74.3)	103 (71.0)	*p* > 0.05
Secondary, technical or higher	26 (25.7)	42 (29.0)	
Type of hospital			
Public	74 (73.3)	107 (73.8)	*p* > 0.05
Private	27 (26.7)	38 (26.2)	
Medical history			
Obesity (≥ 30 kg/m2)	32 (32.3)	33 (23.1)	*p* > 0.05
Hypertension	59 (58.4)	86 (59.3)	*p* > 0.05
Previous myocardial infarction	52 (51.5)	76 (52.4)	*p* > 0.05
Diabetes	32 (31.7)	56 (38.6)	*p* > 0.05
Kidney disease	18 (17.8)	53 (36.6)	0.001
Atrial arrhythmia	27 (26.7)	33 (22.8)	*p* > 0.05
Ventricular arrhythmia	28 (27.7)	8 (5.5)	0.0001
Previous interventional procedures: PTCA/CRM	40 (39.6)	40 (27.6)	0.048
NYHA Stages of Heart Failure			
I	20 (19.8)	25 (17.2)	*p* > 0.05
II	53 (52.5)	76 (52.4)	
III	26 (25.7)	39 (26.9)	
IV	2 (2.0)	5 (3.5)	
Cardiac medical devices			
ICD*	65 (65.6)	0	
CRT-D**	36 (34.4)	0	
CRT-P***	0	25 (17.0)	
Pacemaker	0	21 (14.3)	

*SD *Standard deviation, *PTCA* percutaneous transluminal coronary angioplasty, *CRM* myocardial revascularization surgery. *Implantable cardioverter device (ICD); ** Cardiac resynchronization therapy with defibrillator (CRT-D); *** Cardiac resynchronization therapy with pacemaker (CRT-P)

The analysis by country reveals that patients from Chile had higher implantation rates than those from Mexico: 79/141 (56.0%) vs. 22/105 (21%). Patients from Chile with CIED implanted at baseline or during follow-up were younger than those eligible but without CIED implantation (mean 57.5 ± 12.8 years vs. 64.3 ± 11.6 years, *p* = 0.001). In both countries, patients with ventricular arrhythmia had CIED implanted at baseline or during follow-up more often than those who were eligible but did not receive it (27.8% vs. 6.4% in Chile, 27.3% vs. 4.8% in Mexico). Regarding the analysis of NHA functional classes, in Chile, access to CIED did not significantly differ by NYHA functional class between patients with and without CIED implants. Most of both groups had NYHA II-III classifications. In Mexico, half of the implanted patients had a NYHA class I, and the other half had a NYHA class II. None in NYHA class III received a CIED in Mexico. There were no significant differences based on sex, residence, schooling level, hospital type, medical history, or previous interventions (Table 3).

**Table 3:** Characteristics of patients with heart failure, LVF < = 35%, and ICD or CRT-D by country

Characteristics	Chile, *n* = 141			Mexico, *n* = 105		
	ICD or CRT-D at baseline or implanted during one year of follow-up *n* = 79 (56.0%)	Eligible, but without ICD or CRT-D *n* = 62 (44.0%)	*p* value	ICD or CRT-D at baseline or implanted during one year of follow-up *n* = 22 (21.0%)	Eligible, but without ICD or CRT-D *n* = 83 (79.0%)	*p* value
Age (years), mean (SD)	57.5 (12.8)	64.3 (11.6)	0.0014	62 (15.1)	63.9 (11.4)	*p* > 0.05
	**n (%)**	**n (%)**		**n (%)**	**n (%)**	
Sex						
Female	21 (26.6)	25 (40.3)	*p* > 0.05	2 (9.1)	16 (19.3)	*p* > 0.05
Male	58 (73.4)	37 (59.7)		20 (90.9)	67 (80.7)	
Residence						
Urban	70 (88.6)	53 (85.5)	*p* > 0.05	19 (86.4)	74 (89.2)	*p* > 0.05
Rural ​​	9 (11.4)	9 (14.5)		3 (13.6)	9 (10.8)	
Formal education						
Basic or None	57 (72.2)	40 (64.5)	*p* > 0.05	18 (81.8)	63 (75.9)	*p* > 0.05
Secondary, higher	22 (27.8)	22 (35.5)		4 (18.2)	44 (24.1)	
Type of hospital						
Private	63 (79.6)	56 (90.3)	*p* > 0.05	11 (50.0)	51 (61.5)	*p* > 0.05
Public	16 (20.2)	6 (9.7)		11 (50.0)	32 (38.5)	
Medical history						
Obesity	25 (32.5)	17 (27.9)	*p* > 0.05	7 (31.8)	16 (19.5)	*p* > 0.05
Hypertension	49 (62.0)	39 (62.9)	*p* > 0.05	10 (45.5)	47 (56.6)	*p* > 0.05
Diabetes	24 (30.4)	15 (24.2)	*p* > 0.05	8 (36.4)	41 (49.4)	*p* > 0.05
Kidney disease	12 (15.2)	10 (16.1)	*p* > 0.05	6 (27.3)	43 (51.8)	*p* > 0.05
Previous AMI	39 (49.4)	21 (33.9)	*p* > 0.05	13 (59.1)	55 (66.3)	*p* > 0.05
Atrial arrhythmia	21 (26.6)	19 (30.7)	*p* > 0.05	6 (27.3)	14 (16.9)	*p* > 0.05
Ventricular arrhythmia	22 (27.8)	4 (6.4)	0.001	6 (27.3)	4 (4.8)	0.001
Previous						
interventional procedures PTCA/CRM	35 (44.3)	19 (30.7)	*p* > 0.05	5 (22.7)	21 (25.3)	*p* > 0.05
NYHA Stages of Heart Failure						
I	9 (11.4)	6 (9.7)	*p* > 0.05	11 (50.0)	19 (22.9)	*p* > 0.05
II	43 (54.4)	29 (46.8)		10 (45.4)	47 (56.6)	
III	26 (32.9)	26 (41.9)		0.0	13 (15.7)	
IV	1 (1.3)	1 (1.6)		1 (4.6)	4 (4.8)	
Cardiac medical devices						
ICD	48 (60.7)	0		17 (77.3)	0	
CRT-D	31 (39.2)	0		5 (22.7)	0	
CRT-P	0	22 (35.4)		0	3 (3.6)	
Pacemaker	0	9 (14.5)		0	12 (14.4)	
No cardiac device	0	31(50.0)		0	68 (82.0)	

*SD* Standard deviation, *PTCA* percutaneous transluminal coronary angioplasty, *CRM* myocardial revascularization surgery. *Implantable cardioverter device (ICD); ** Cardiac resynchronization therapy with defibrillator (CRT-D); *** Cardiac resynchronization therapy with pacemaker (CRT-P)

The analysis of reasons for not receiving an ICD/CRT-D among eligible men and women in both countries indicates that, in Chile, the primary reason was having received a pacemaker or CRT-P instead (up to 56% of Chilean women and 46% of men). This was followed by individuals being on the waiting list, under evaluation, physicians not prescribing a CIED, death during follow-up, or other reasons, such as financial barriers to transportation. In Mexico, the leading cause was being on the waiting list for an implant (37.5% of women and 22.4% of men), followed by being under evaluation, or because medical doctors did not prescribe the CIED. No statistically significant differences in the reasons for not receiving an ICD or a CRT-D were observed between men and women in either country (Table 4).

**Table 4:** Reasons for not receiving ICD or CRT-D by country, separated by sex

	Chile			Mexico		
	Men	Women		Men	Women	
	n=37 (%)	n=25 (%)		n=67 (%)	n=16 (%)	
Patients with PM or CRT_P	17 (46.0)	14 (56.0)	*p* >0.05	12 (17.9)	2 (12.5)	*p* >0.05
Patients with CIED prescription on waiting list for implant	7 (19.0)	2 (8.0)	*p* >0.05	15 (22.4)	6 (37.5)	*p* >0.05
Patients under evaluation for CIED implant approval	3 (8.1)	4 (16.0)	*p* >0.05	17 (25.4)	4 (25.0)	*p* >0.05
Medical doctors did not prescribe ICD or CRT-D	2 (5.4)	2 (8.0)	*p* >0.05	25 (22.4)	3 (18.8)	*p* >0.05
Death during the follow-up	3 (8.1)	0	*p* >0.05	3 (4.5)	0	*p* >0.05
Other (e.g., patients cannot afford the cost to attend the procedure)	5 (13.5)	3 (12.0)	*p* >0.05	5 (7.5)	1(6.3)	*p* >0.05

The multivariate analysis, including data from Chile and Mexico, reveals that, after adjusting for sociodemographic and clinical variables and accounting for patients clustering in hospitals, several factors were independently associated with the likelihood of not receiving an ICD or CRT-D. Patients from Mexico had 7.6 times the odds of not receiving a device compared to those from Chile (adjusted OR 7.58, 95% CI 3.21–17.91; *p* < 0.001). Hospital type: care in a public hospital increased the odds nearly fourfold (aOR 3.87, 95% CI 2.20–6.80; *p* < 0.001). Age: Each additional year of age increased the odds of non-implantation by 5% (aOR 1.05, 95% CI 1.01–1.09; *p* = 0.02). NYHA class: Patients in NYHA III–IV had higher odds of not receiving a device than those in NYHA I (aOR 5.28, 95% CI 1.82–15.28; *p* = 0.002). The presence of ventricular arrhythmia was strongly associated with a lower likelihood of non-implantation (aOR 0.09, 95% CI 0.04–0.18; *p* < 0.001), as well as a history of revascularization (aOR 0.57, 95% CI 0.33–0.99; *p* = 0.047). Sex, education, comorbidities, and rural residence were not significantly associated with access and were included only to adjust for potential confounders. No evidence of multicollinearity or significant interactions was found. The model had a good fit (Hosmer–Lemeshow *p* > 0.05; pseudo-R² = 0.2652) (Table 5).

**Table 5:** Factors associated with the likelihood of not receiving ICD or CRT-D devices among patients with heart failure and LVEF < = 35% in Chile and Mexico

Covariates	Adjusted OR	95% Confidence intervals	*p*
Age (years)	1.05	1.007, 1.09	0.020
Male sex	0.99	0.28, 3.53	0.997
None or basic formal education	0.74	0.33, 1.66	0.466
Mexico	7.58	3.21, 17.91	0.0001
Rural residence	1.38	0.57, 3.35	0.478
Public hospital	3.87	2.20, 6.80	0.0001
Medical history			
Obesity (≥ 30 kg/m2)	0.68	0.31, 1.50	0.341
Hypertension	1.06	0.66, 1.72	0.870
Previous myocardial infarction	0.64	0.31, 1.35	0.241
Diabetes	0.60	0.32, 1.10	0.096
Kidney disease	1.99	0.57, 6.99	0.281
Atrial arrhythmia	0.61	0.31, 1.23	0.167
Ventricular arrhythmia	0.09	0.04, 0.18	0.0001
Previous interventional procedures: PTCA/CRM	0.57	0.33, 0.99	0.047
NYHA Stages of Heart Failure			
II	2.30	0.74, 7.18	0.151
III-IV	5.28	1.82, 15.28	0.002

*PTCA* percutaneous transluminal coronary angioplasty, *CRM* myocardial revascularization surgery

The country-specific sensitivity analysis confirmed that public hospitals were linked to higher odds of not receiving ICD or CRT-D devices in both countries. In contrast, increased age was especially significant in Chile and among patients with NYHA Heart Failure Stages III and IV in Mexico. There were no sex-related disparities in receiving or in the reasons for not receiving ICD or CRT-D devices (Supplementary Tables 1 and 2).

When examining factors associated with both no device and alternative device (pacemaker or CRT-P) implantation, using guideline-concordant ICD/CRT-D implantation as the reference, we found results that were generally consistent with the main findings. Receiving care in public hospitals, residence in Mexico, and NYHA class III–IV were associated with higher odds of not receiving an ICD/CRT-D device. However, age was only associated with a higher likelihood of pacemaker or CRT-P implantation, whereas a history of revascularization and ventricular arrhythmia reduced these odds (see Supplementary Table 3).

## Discussion

This research underscores disparities between Chile and Mexico in access to and appropriate prescription of CIED. The access gaps in both countries were tangible. In Chile, 44% of eligible patients did not receive an ICD or a CRT-D, whereas in Mexico, the proportion was 79% during the follow-up year. Key predictors associated with access barriers included living in Mexico, treatment at public facilities, older age, and higher NYHA class. Patients with a clear indication, such as ventricular arrhythmia and a history of previous revascularization, were more likely to receive a CIED, aligning with the prioritization criteria. No significant sex-related disparities in access to ICD or CRT-D were observed. In Chile, most eligible or implanted patients were classified as NYHA II–III, consistent with guideline indications. In Mexico, 50% of implanted patients had a NYHA I classification, which is unusual, as devices are typically recommended for patients with NYHA II–III classifications. No devices were provided to patients with NYHA III.

These findings should be interpreted within the broader context of evolving heart failure management. Contemporary evidence suggests that optimized guideline-directed medical therapy may reduce mortality, arrhythmic burden, and the need for device therapies. A recent meta-analysis highlights the growing role of modern pharmacological therapies in modifying arrhythmic risk and reinforces the importance of integrating medical and device-based strategies [25].

### Access barriers to CIEDs have been linked to structural factors in healthcare

Healthcare services require improved access to and appropriate prescribing of CIEDs for HF patients, particularly in public health settings. Various studies have examined the capacity of public healthcare facilities to serve HF patients across regions, including China [26], Europe [27], and sub-Saharan Africa [28]. These investigations involved substantial patient cohorts, hospitals, and countries. The results showed that resource density was higher in countries endowed with greater financial, human, and technical resources. In our study, 4 of 10 patients in Chile and 8 of 10 in Mexico did not receive a CIED. Among patients receiving care at public health facilities, the likelihood of not receiving a CIED was nearly 4 times that in private settings. In Chile, the AUGE/GES plan covers several cardiovascular conditions, such as acute myocardial infarction (AMI), arrhythmias, and chronic issues with the aortic valve explicitly. These conditions can lead to HF. Previous evaluations of AUGE/GES reported that the plan improved health outcomes for patients with AMI [29]. In Mexico, people lacking social security health benefits receive care at Ministry of Health facilities, although they do not have an explicit package of health benefits. For patients with chronic diseases, the probability of receiving medications and devices is uncertain, given the scarcity of resources in Mexico’s health sector. This is driven by financial, geographic, and systemic barriers, with rural populations in vulnerable situations being the most affected [30].

### Notable disparities were observed in adherence to the 2017 ACC/AHA/HRS Class I criteria between Chile and Mexico

In Chile, most patients with implantable devices or those eligible for implantation were classified as NYHA class II or III, consistent with guideline-recommended indications. Conversely, in Mexico, 50% of patients with implanted devices were classified as NYHA class I, an uncommon observation given that according to the 2017 ACC/AHA/HRS Class I guidelines recommendations device prescription typically targets patients with NYHA class II–III, with stricter criteria for NYHA I (such as Left Bundle Branch Block, ≥ 40 days post-myocardial infarction, or QRS ≥ 150 ms with optimal therapy). Yet, no patients classified as NYHA III received devices in Mexico, despite this group being the most indicated. This pattern suggests that other factors, rather than pre-existing comorbidities, contribute to the access discrepancy; it also implies barriers to access or delayed referrals for symptomatic NYHA III patients within Mexican public hospitals. A cohort study involving 37,745 patients identified gaps in evidence-based knowledge to guide CIED selection, recognizing that clinicians consider different variables when prescribing these devices. Such findings justify further research to assess the appropriateness and quality of initial indications for device implantation [31].

### Individual-related factors were associated with lower access to CIED

Older age of patients and living in Mexico were associated with lower access to CIED. Each additional year of age increased the odds of non-implantation by 5%, and patients living in Mexico had 7.6 times higher odds of not receiving the device. This finding is not uncommon. The use of CIEDs among eligible HF patients ranges from 1.5% in Indonesia to 52.5% in Japan, with higher uptake in regions with stronger government reimbursement and financial protection policies [32].

### There were no sex-related disparities regarding access to CIEDs

Across both countries, no statistically significant difference in device access was observed between men and women after adjustment for covariates (aOR 0.99, 95% CI 0.28–3.53; *p* = 0.997). However, the relatively small sample size of women (*n* = 64) limits statistical power, and the observed effect direction suggests that potential sex differences cannot be excluded. The importance of gaining in-depth knowledge of sex differences is justified. A study conducted in Chile reported the differences in the incidence and mortality of heart failure among women and men [33]. While the incidence of heart failure hospitalizations was higher in men and increased significantly with age, the prevalence of hospitalizations was higher in women, who also experienced a greater risk of in-hospital mortality. However, this effect was no longer evident after adjusting for age in the multivariable model [33]. A retrospective cohort study from Australia (2008–2018) showed that women with arrhythmia, cardiomyopathy, or syncope were less likely to receive pacemakers, defibrillators, or resynchronization therapy [34]. A similar pattern was observed in the United States, where female gender was independently linked to lower rates of ICD and device implantation [35].

### Limitations

Due to the secondary data analysis nature of this study, several limitations should be considered. First, comparisons between countries involved unequal sample sizes and participant heterogeneity, which may lead to overinterpretation of the results. Second, the small number of female participants could affect the definitive conclusion regarding the absence of sex-related differences. Third, the sample sizes for country-specific sensitivity analyses were relatively small, resulting in wide confidence intervals and increasing the risk of Type II errors, which might cause us to miss true associations within each country. Fourth, the associations observed in this study are based on observational data and should therefore be interpreted as hypothesis-generating rather than causal. Residual confounding and unmeasured clinical factors may influence both eligibility and access to device implantation. Lastly, the generalizability of the findings may be limited because the study focused only on two Latin American countries and used non-probability sampling, with 34–50 consecutive patients recruited per hospital. However, these patients represented real-world cases with multiple comorbidities, accurately reflecting the clinical conditions typically seen in routine practice in these countries.

## Conclusion

Patients with heart failure in Chile and Mexico encounter obstacles in accessing cardiac implantable devices, reducing their chances of improved health outcomes. These barriers relate to factors like older age, country of residence, and treatment in public hospitals. No significant sex differences in access to CIED were found. Notably, implantation rates for ICDs and CRT-Ds were significantly higher in Chile than in Mexico, and compared to Chile, Mexico had 4 times more recipients with NYHA class I, even though the 2017 ACC/AHA/HRS Class I guidelines recommend ICDs and CRT-Ds mainly for symptomatic NYHA II–III patients, with stricter criteria for NYHA I (such as Left Bundle Branch Block, ≥ 40 days post-myocardial infarction, or QRS ≥ 150 ms with optimal therapy).

These findings highlight inequities in access to CIEDs in both countries and underscore the need for addressing multiple interconnected barriers beyond simple resource availability. While financial constraints and limited infrastructure—particularly in public healthcare systems—play a key role, our findings suggest that variability in clinical decision-making also contribute substantially to underutilization. Strengthening explicit financing mechanisms, expanding the number of implanting centers and trained specialists, and improving integration across levels of care are critical steps. In line with contemporary recommendations, optimizing the timing and appropriate use of devices within structured heart failure programs is essential to ensure that eligible patients receive these therapies in a timely and equitable manner.

## Supplementary Information

Supplementary Material 1.

## Data Availability

The database of the study is available upon reasonable request from Judith Riesgo: judith.riesgo@medtronic.com.

## Declarations

### Clinical trial number

Not applicable, as this is a secondary data analysis of the observational study.

## Abbreviations

ACC/AHA/HRS: American Heart Association, the American College of Cardiology, and the Heart Rhythm Society
CIEDs: Cardiac implantable devices
CVD: Cardiovascular diseases
CRT-Ds: Cardiac resynchronization therapy with defibrillator
CRT-P: Pacemaker-based cardiac resynchronization therapy
CABG: Coronary artery bypass grafting
ESC: The European Society of Cardiology
HF: Heart failure
ICDs: Implantable cardioverter defibrillators
LAC: Latin America and the Caribbean
LVEF: Left ventricular ejection fraction
MI: Myocardial infarction
NYHA: New York Heart Association Functional Classification
PLASMA: Probability of Suffering Arrhythmic Death
PM: Pacemakers
