# Supplementary material for: Inequities in access to life-saving cardiac devices for heart failure: a comparative analysis of Chile and Mexico
Source: BMC Cardiovasc Disord. 2026 May 11;26:567. doi: 10.1186/s12872-026-05924-4 (PMC13335306; doi:10.1186/s12872-026-05924-4)
Supplement: Supplementary file 1 — Supplementary Material 1. [file 12872_2026_5924_MOESM1_ESM.pdf]

Supplementary material.

Supplementary table 1. Factors associated with the probability of not receiving ICD or CRT-D devices by patients with heart failure and LVEF<=35% in Chile

	**Adjusted OR**	**95% Confidence intervals**	p
Age (years)	**1.08**	**1.02, 1.14**	**0.010**
Male sex	0.87	0.18, 4.19	0.867
None or basic formal education	0.67	0.28, 1.58	0.359
Rural residence	1.40	0.40, 4.84	0.598
**Public hospital**	**3.08**	**2.09, 4.53**	**0.0001**
Medical history			
Obesity (≥30 kg/m2)	0.84	0.33, 2.16	0.721
Hypertension	0.65	0.32, 1.30	0.225
Previous myocardial infarction	0.56	0.24, 1.30	0. 771
Diabetes	0.59	0.20, 1.74	0.338
Kidney disease	1.33	0.70, 2.54	0.385
Atrial arrhythmia	0.88	0.31, 1.23	0.765
**Ventricular arrhythmia**	**0.09**	**0.05, 0.18**	**0.0001**
Previous interventional procedures: PTCA/CRM	0.64	0.32, 1.26	0.197
NYHA II-III-IV	1.12	0.38, 3.25	0.838
**Reference categories**: female sex, secondary or technical or higher, urban residence, private hospital, without obesity, without medical diagnosis of; hypertension, diabetes, or kidney disease, without previous myocardial infarction, without atrial arrhythmia, or ventricular arrhythmia, without previous PTCA or CRM, NYHA I.			

Supplementary table 2. Factors associated with the probability of not receiving ICD or CRT-D devices by patients with heart failure and LVEF<=35% in México

	**Adjusted OR**	**95% Confidence intervals**	p
Age (years)	1.002	0.96, 1.05	0.919
Male sex	0.50	0.04, 6.58	0.599
None or basic formal education	1.09	0.09, 12.64	0.943
Rural residence	0.76	0.15, 3.89	0. 747
**Public hospital**	**2.70**	**1.19, 6.13**	**0.018**
Medical history			
Obesity (≥30 kg/m2)	0.37	0.09, 1.54	0.173
Hypertension	1.26	0.37, 4.16	0.733
Previous myocardial infarction	1.02	0.33, 3.19	0.972
Diabetes	0.82	0.35, 1.91	0. 649
Kidney disease	3.80	0.36, 39.98	0.265
Atrial arrhythmia	0.43	0.08, 2.49	0.350
**Ventricular arrhythmia**	**0.09**	**0.02, 0.58**	**0.010**
Previous interventional procedures: PTCA/CRM	0.89	0.50, 1.60	0.709
**NYHA II-III-IV**	**6.14**	**3.16, 11.96**	**0.0001**

Reference categories: female sex, secondary or technical or higher, urban residence, private hospital, without obesity, without medical diagnosis of; hypertension, diabetes, or kidney disease, without previous myocardial infarction, without atrial arrhythmia, or ventricular arrhythmia, without previous PTCA or CRM, NYHA I.

Supplementary table 3. Factors associated with the probability of not receiving ICD or CRT-D devices by patients with heart failure and LVEF<=35% and alternative device (pacemaker or CRT-P) implantation

Covariates	Adjusted OR	95% Confidence intervals	p
**Factors associated with the likelihood of not receiving ICD or CRT-D devices**			
Age (years)	1.03	0.98, 1.07	0.221
Male sex	1.02	0.28, 3.75	0.971
None or basic formal education	0.70	0.32, 1.43	0.370
**Mexico**	**10.55**	**4.08, 27.23**	**0.0001**
Rural residence	1.34	0.38, 4.74	0.647
**Public hospital**	**3.09**	**1.79, 5.33**	**0.0001**
Medical history			
Obesity (≥30 kg/m2)	0.75	0.32, 1.77	0.507
Hypertension	1.11	0.67, 1.85	0.674
Previous myocardial infarction	0.74	0.36, 1.54	0.422
Diabetes	0.68	0.35, 1.35	0.271
Kidney disease	1.97	0.50, 7.78	0.333
**Atrial arrhythmia**	**0.41**	**0.20, 0.84**	**0.015**
**Ventricular arrhythmia**	**0.09**	**0.34, 0.25**	**0.0001**
Previous interventional procedures: PTCA/CRM	0.73	0.37, 1.44	0.362
NYHA Stages of Heart Failure			
II	2.30	0.76, 6.97	0.140
**III-IV**	**5.00**	**1.37, 18.15**	**0.015**
**Factors associated with the likelihood of alternative device (pacemaker or CRT-P) implantation**			
Age (years)	**1.11**	**1.07, 1.16**	**0.0001**
Male sex	0.90	0.23, 3.56	0.877
None or basic formal education	0.71	0.22, 2.27	0.567
Mexico	2.58	0.66, 10.14	0.174
Rural residence	1.21	0.29, 4.97	0.795
**Public hospital**	**6.31**	**1.42, 28.04**	**0.016**
Medical history			
Obesity (≥30 kg/m2)	0.60	0.19, 1.85	0.376
Hypertension	0.85	0.43, 1.66	0.628
Previous myocardial infarction	0.59	0.21, 1.64	0.308
Diabetes	0.46	0.19, 1.09	0.078
Kidney disease	2.32	0.84, 6.46	0.106
Atrial arrhythmia	1.12	0.51, 2.42	0.781
**Ventricular arrhythmia**	**0.07**	**0.02, 0.22**	**0.0001**

**Covariates**	**Adjusted OR**	**95% Confidence intervals**	p
**Factors associated with the likelihood of not receiving ICD or CRT-D devices**			
Previous interventional procedures: PTCA/CRM	**0.37**	**0.20, 0.71**	**0.003**
NYHA Stages of Heart Failure			
II	3.18	0.71, 14.31	0.132
**III-IV**	**6.77**	**2.19, 20.92**	**0.001**
